# The Two-Year Itch: Extreme Body Lice Infestation Complicated by Anemia, Eosinophilia, and Elevated Immunoglobulin E

**DOI:** 10.7759/cureus.87251

**Published:** 2025-07-03

**Authors:** George S Zacharia, Shivani Jani, Satyam Mahaju, Manjola Doda, Neelanjana Pandey, Sabirah Kasule, Yudhistra Persaud, Donald Rudikoff

**Affiliations:** 1 Internal Medicine, BronxCare Health System, New York, USA; 2 Infectious Diseases, BronxCare Health System, New York, USA; 3 Allergy and Immunology, BronxCare Health System, New York, USA; 4 Dermatology, BronxCare Health System, New York, USA

**Keywords:** anemia, body lice, eosinophilia, immunoglobulin e, pediculus

## Abstract

Human lice infestation, or pediculosis, remains a significant global public health concern, particularly in vulnerable populations living in overcrowded, poorly hygienic conditions. Lice are known to be vectors for arthropod-borne diseases such as epidemic typhus and trench fever. However, a growing body of evidence suggests their potential role in the pathogenesis of iron deficiency anemia, peripheral eosinophilia, and elevated immunoglobulin E (IgE) levels. We report a case of a middle-aged male with a two-year history of extensive pruritic skin lesions in the setting of a visible lice infestation over his trunk and clothes. Laboratory investigations demonstrated iron deficiency anemia, peripheral eosinophilia, and markedly elevated serum IgE levels. The patient was treated with topical permethrin and oral ivermectin, along with antibiotics for secondary infection. The infested clothing and other belongings were appropriately discarded to prevent reinfestation. The patient’s skin lesions improved, as did his anemia and eosinophilia, suggesting a causal association between the chronic louse infestation and the observed laboratory derangements.

## Introduction

Hematophagous ectoparasites of the genus *Pediculus* and *Pthirus* are responsible for human pediculosis. Head lice infections, caused by *Pediculus humanus capitis*, are more frequent in primary school children, with girls having nearly 3.5 times the risk than boys [1,2]. On the other hand, body lice infections are caused by *Pediculus humanus humanus*. They are more common in adults and are associated with poor hygiene, overcrowding, and lack of clean clothes [3]. Finally, *Pthirus pubis*, also known as pubic lice, is typically sexually transmitted and is the least common cause of lice infestation [4]. Ectoparasites are not typically regarded as a frequent cause of peripheral eosinophilia, with a possible exception of severe infestations [5,6]. Furthermore, the literature suggests that these severe infestations can also be associated with iron deficiency anemia, presumably due to blood loss [7-9]. Our patient, an undomiciled middle-aged male, presented to us with extensive pruritic skin lesions and a severe body lice infestation. His presentation was complicated by eosinophilia and anemia, as well as significantly elevated immunoglobulin E (IgE) levels. We discuss management considerations in this patient, as well as an etiological link between the lice infestation and his hematological and immunological abnormalities.

## Case presentation

A 51-year-old male with a history of schizophrenia and asthma presented for a two-year history of pruritus, which had progressed in the last couple of weeks with the development of extensive skin excoriations, erosions, and scattered pustules, a few with exuding pus. He denied any constitutional symptoms, had no recent travel, and did not take any medications. He was unemployed and unhoused. Most recently, he lived with a friend under poor hygienic conditions. He was sexually active but denied recent exposure or similar lesions in his partners. 

**Figure 1:**
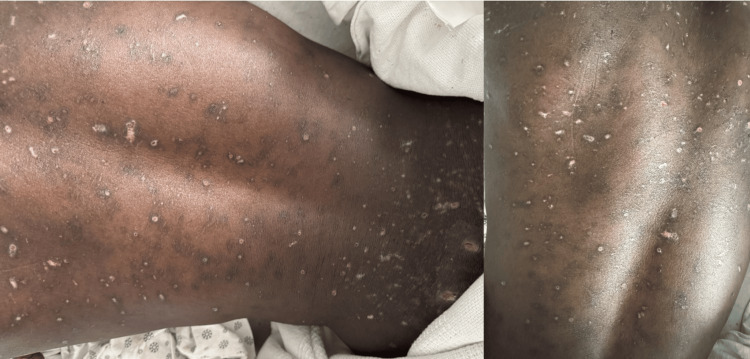
Extensive skin lesions along the back of the trunk with skin erosions, superficial healing ulcerated lesions, and areas of likely post-inflammatory hyperpigmentation.

At presentation, he was disheveled and uncomfortable, but was hemodynamically stable. Examination revealed multiple pustules and crusted, ulcerated lesions, most prominent on the back, chest, arms, and thighs, with several areas of hyperpigmentation (Figure 1).

**Figure 2:**
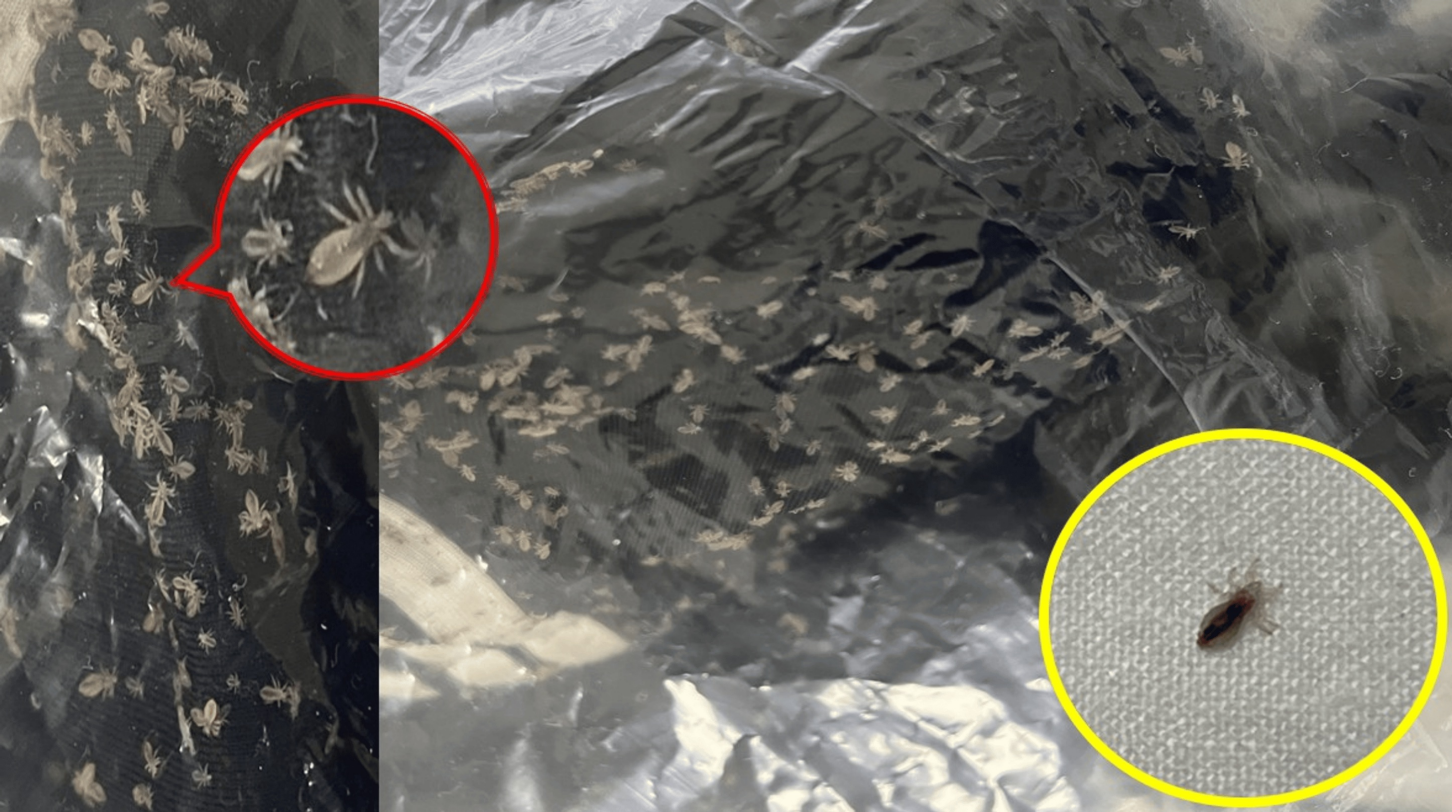
Multiple lice infest the patient's fomites, including clothes and bags, which are sealed off in transparent bags. Magnified view of pale lice devoid of hematophagy (red circle). Image of the lice on the patient's bed appearing darker following a blood meal (yellow circle).

Few lice were detected over his body and bed, but his bags and clothes teemed with live lice (Figure 2). Retrospectively, the patient reported noticing the bugs for the last two years but never sought treatment. He had only come this time because of the intense pruritus and pustular lesions.

Labs were significant for microcytic hypochromic anemia, severe eosinophilia (1,600 cells/μL), thrombocytosis, elevated inflammatory markers, and elevated IgE levels at 9,820 kU/L (normal range <114 kU/L). The peripheral blood film revealed a microcytic hypochromic picture, with anisopoikilocytosis and eosinophilia. Further evaluation was consistent with hypo-proliferative anemia due to iron deficiency (Table 1).

**Table 1:** An excerpt of the initial hematological and biochemical evaluation. MCV: mean copuscular volume; CRP: C-reactive protein; ESR: erythrocyte sedimentation rate; IgE: immunoglobulin E; ESR: erythrocyte sedimentation rate; UIBC: unsaturated iron binding capacity

Parameter	Results			Reference range
	Day 0	Day 5	Day 14	
Hemoglobin	9.9	10.3	10.9	12-16 g/dl
MCV	72.8	--	--	80-96 fL
Leukocyte count	10.2	10.7	5.9	4.8-10.8 k/ul
Eosinophil count	1.6	1.1	0.7	0.05-0.25 k/uL
Platelets	483	503	462	150-400 microL
ESR	101.0	--	--	≤20 mm/hr
CRP	67.36	57.49	8.3	≤5.0 mg/L
Iron	16	--	--	65-175 ug/dl
Ferritin	23.6	--	--	13-150 ng/ml
UIBC	237	--	--	112-346 ug/dl
Vitamin B12	463	--	--	243-894 pg/ml
Folic acid	10.7	--	--	>5.4 ng/ml
IgE	9820	--	8630	≤114 kU/L
TSH	2.34	--	--	0.26-2.66 mIU/L

**Figure 3:**
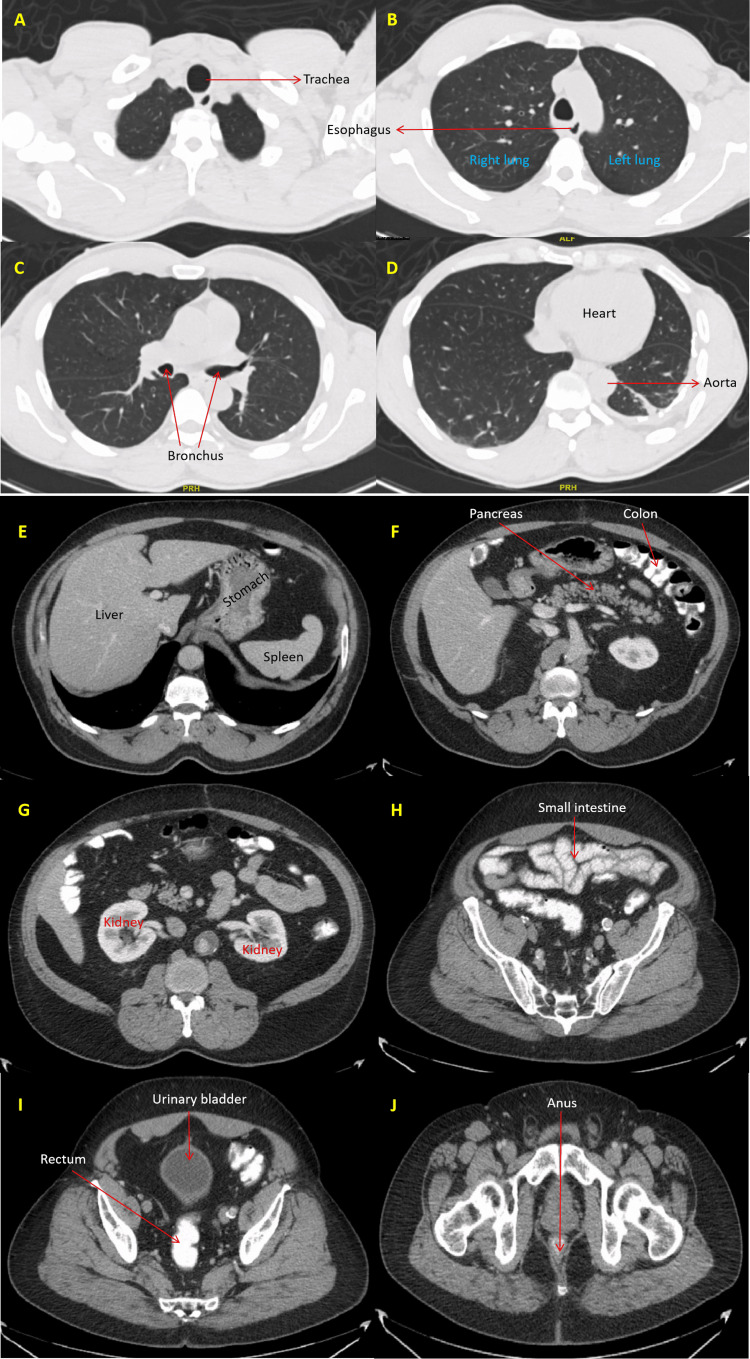
Computerized tomography (CT) images. A to D: Axial images of the chest revealed normal tracheobronchial tree, lungs, heart, aorta and esophagus. E to J: Axial abdominal images depicting normal hollow and solid visceral organs.

The patient was immediately isolated and empirically given topical permethrin, mupirocin, and intravenous vancomycin. His clothes and belongings were disposed of safely. Cultures from the discharge of his wounds isolated methicillin-resistant *Staphylococcus aureus* (MRSA). Other infectious workups, including blood cultures; screening for human immunodeficiency virus; polymerase chain reaction (PCR) swabs of the wounds for varicella-zoster virus, herpes simplex virus (HSV) 1, HSV 2, and Mpox virus; rapid plasma reagin for syphilis; urine nucleic acid amplification test for gonorrhea and chlamydia, *Strongyloides stercoralis* IgG; and stool analysis for ova and parasites and *Helicobacter pylori*, were negative (Table 2). Furthermore, fecal calprotectin and occult blood were negative. He declined bidirectional endoscopies for the evaluation of anemia. Celiac disease antibodies were undetectable. Computed tomography of the chest and abdomen did not reveal any clinically significant findings (Figure 3).

**Table 2:** A summary of relevant infectious diseases and anemia evaluation. PCR: polymerase chain reaction; DNA: deoxyribonucleic acid; MRSA: methicillin-resistant* Staphylococcus aureus*

Parameter	Results	Reference range
Monkeypox virus, DNA, PCR	Not detected	Not detected
Orthopox virus, DNA, PCR	Not detected	Not detected
Varicella Zoster virus, DNA, PCR	Not detected	Not detected
Hepes Simplex virus 1 & 2, DNA, PCR	Not detected	Not detected
Human Immunodeficiency virus 1 & 2	Non reactive	Non-reactive
Swab culture (skin lesions)	Light growth of MRSA	Not detected
Aspergillus antigen	Not detected	Not detected
Blastomyces antibodies	Not detected	Not detected
Coccidioides antibodies	Negative	Negative
Cryoglobulin, qualitative	Negative	Negative
Strongyloides antibodies	Negative	Negative
β-D-Glucan	Negative	Negative
Hepatitis B surface antigen	Negative	Negative
Hepatitis C antibodies	Negative	Negative
IgA tissue transglutaminase	Negative	Negative
Stool ova, cysts, parasites	Not detected	Not detected
Stool occult blood	Negative	Negative
Fecal calprotectin	13	

For the elevated IgE, Allergy and Immunology were consulted, and they determined that while the patient did have a history of concurrent mild intermittent asthma, it is unlikely to explain the high levels of eosinophilia or IgE, considering that the patient had no active symptoms or exacerbations currently or in the recent past, despite being off medications for a long duration. They agreed that it is highly likely that the severe ectoparasitic infection is driving his immunological phenomena, as his allergy testing also failed to explain his manifestations. 

During the hospital course, the patient had decompensation of his underlying schizophrenia and was transferred to an inpatient psychiatric facility. He received another course of topical 1% permethrin. He completed a seven-day course of mupirocin and vancomycin, as well as two courses of topical permethrin, spaced one week apart. His skin findings significantly improved, as did his eosinophilia and anemia. He is scheduled for an outpatient review for a follow-up assessment of his IgE levels, as a drop in IgE levels typically occurs weeks to months after an allergic reaction.

## Discussion

Human lice are blood-feeding, wingless arthropods belonging to the Pediculus or Pthirus family. Paleoentomological studies suggest lice are the oldest known human ectoparasites, with Brazilian archaeological studies demonstrating louse nits in 10,000-year-old human hair samples [10]. Poor hygiene, overcrowding, homelessness, and lack of access to clean clothes are linked to corporal pediculosis [3]. *Pediculus humanus*, or body lice, specifically inhabit clothing and move to the body for feeding. Transmission of lice from person to person occurs through direct contact or the sharing of infested clothing or belongings. The lice move from one area to another by crawling and cannot fly or jump. In addition, animals or pets are not known to carry or spread lice [11]. Humans are the only known hosts of lice that exclusively feed on human blood.

Pruritus, the hallmark clinical feature of lice infestation, results from an allergic or inflammatory reaction to the lice secretions [3,12]. During the initial exposure, there may be no itching, as sensitization is required for the immunological response [12]. Given this known immunologic response, it is unsurprising that lice infestation could be linked to significant elevations in IgE. Fernandez et al. demonstrated IgE-mediated hypersensitivity reactions to head lice, resulting in recurrent asthma exacerbations [6]. Their patient recovered completely once his head lice were eradicated. Other studies regarding allergy to lice do not discuss IgE levels, except for one by Pagnapapplou et al. [13]. However, their patients' IgE elevations were not as high as ours. Interestingly, this patient had eosinophilia, just like our patient. Animal studies have reported eosinophilia associated with lice infestation [14]. Cases of eosinophilia secondary to human lice infestations are reported in the published literature [5,13]. The hypersensitivity reaction to ectoparasite salivary proteins is hypothesized to contribute to allergic responses and eosinophilia. Eosinophilia typically regresses once the lice infestation is resolved.

A heavy lice infestation has been linked to iron deficiency anemia [7-9]. Speare et al. reported blood draws of 0.0001579 ml, 0.0000657 ml, and 0.0000387 ml with each bite from an adult female, adult male, and nymph of Pediculus capitis, respectively [15]. In heavily infested individuals, provided the lice feed 4 to 5 times a day, this can predispose to noticeable blood loss. In a healthy individual, the body produces blood exceeding the imbibing potential of thousands of lice. However, when combined with multiple minor blood losses related to excoriation from intense itching and poor nutritional status, heavy lice infestation can exacerbate iron deficiency anemia [5,7].

Our patient presented with a heavy lice infestation, eosinophilia, anemia, and elevated IgE. Separately, the eosinophils may have been an exaggerated response to atopy, and the anemia was nutritionally mediated. Still, with improvement in his lice infestation, he improved his anemia and eosinophilia. The prompt hematological response to lice eradication suggests that, at the very least, the lice were exacerbating his underlying conditions if not causing them. Also, the level of IgE elevation was too high for the level of atopy he had, and he did not meet the criteria for Job syndrome. These elevated IgE levels were still elevated at the repeat test just before discharge, but it is expected that they will take time to improve.

Peripheral eosinophilia, defined as an absolute eosinophil count greater than 500/µL, has a broad differential diagnosis. The most common cause worldwide is parasitic infection, particularly helminths such as Strongyloides, Ascaris, Toxocara, and Schistosoma species. Allergic and atopic conditions like asthma, allergic rhinitis, eczema, and drug hypersensitivity reactions are frequent offenders in developed countries. Hematologic malignancies, including chronic eosinophilic leukemia, Hodgkin's lymphoma, and certain T-cell lymphomas, as well as autoimmune disorders such as eosinophilic granulomatosis with polyangiitis, and connective tissue diseases, are uncommon causes of eosinophilia. Other causes include eosinophilic gastrointestinal disorders, adrenal insufficiency, pulmonary eosinophilic syndromes, and idiopathic hypereosinophilic syndrome [16]. 

## Conclusions

Human lice infestations are associated with pruritic skin diseases, secondary bacterial skin infections, and the transmission of diseases such as epidemic typhus, relapsing fever, and trench fever. Rarely has an association with iron deficiency anemia, eosinophilia, or elevated IgE levels been reported. Clinicians should maintain vigilance for ectoparasitic infections as an underlying etiology of otherwise unexplained iron deficiency or allergic reactions, as complete resolution of this condition requires treatment of the lice and addressing the other exacerbating factors.
